# Interaction of perceived social support and childhood maltreatment on limbic responsivity towards negative emotional stimuli in healthy individuals

**DOI:** 10.1038/s41386-024-01910-6

**Published:** 2024-07-01

**Authors:** Tiana Borgers, Anne Rinck, Verena Enneking, Melissa Klug, Alexandra Winter, Marius Gruber, Anna Kraus, Katharina Dohm, Elisabeth J. Leehr, Dominik Grotegerd, Katharina Förster, Janik Goltermann, Jochen Bauer, Udo Dannlowski, Ronny Redlich

**Affiliations:** 1 Institute for Translational Psychiatry, University of Münster, Münster, Germany; 2 Department of Psychiatry, Psychosomatic Medicine and Psychotherapy, University Hospital Frankfurt, Goethe University, Frankfurt, Germany; 3 Clinical Psychology and Behavioral Neuroscience, Faculty of Psychology, Technische Universität Dresden, Dresden, Germany; 4 Department of Clinical Radiology, University of Münster, Münster, Germany; 5 Department of Psychology, University of Halle, Münster, Germany; 6 German Center for Mental Health (Deutsches Zentrum für Psychische Gesundheit), Halle, Germany; 7 Center for Intervention and Research on adaptive and maladaptive brain circuits underlying mental health (C-I-R-C), Halle, Germany

**Keywords:** Risk factors, Limbic system

## Abstract

Childhood maltreatment (CM) is associated with increased limbic activity, while social support is linked to decreased limbic activity towards negative stimuli. Our study aimed to explore the interaction of perceived social support with CM, and their combined impact on limbic activity in negative emotion processing. A total of 130 healthy individuals (HC) underwent a negative emotional face processing paradigm. They were divided into two groups based on the Childhood Trauma Questionnaire: *n* = 65 HC without CM matched with *n* = 65 HC with CM. In a region-of-interest approach of the bilateral amygdala-hippocampus-complex (AHC), regression analyses investigating the association of CM and perceived social support with limbic activity and a social support x CM ANCOVA were conducted. CM was associated with increased AHC activity, while perceived social support tended to be associated with decreased AHC activity during negative emotion processing. The ANCOVA showed a significant interaction in bilateral AHC activity (*p*_*FWE*_ ≤ 0.024) driven by a negative association between perceived social support and bilateral AHC activity in HC without CM. No significant association was observed in HC with CM. Exploratory analyses using continuous CM scores support this finding. Our results suggest that CM moderates the link between perceived social support and limbic activity, with a protective effect of perceived social support only in HC without CM. The lack of this effect in HC with CM suggests that CM may alter the buffering effect of perceived social support on limbic functioning, highlighting the potential need for preventive interventions targeting social perception of HC with CM.

## Introduction

Perceived social support is a key protective factor, enhancing physical and psychological health, mitigating disease progression, and reducing mortality [1]. It is associated with lower allostatic load [2] and diminished stress responses [3]. The perception of having a supportive social network seems to buffer physiological stress responses to critical life events [4, 5**]**, potentially increasing resilience against mental health issues, particularly following childhood maltreatment (CM) [6]. Conversely, social conflict can impair wellbeing, especially in those with insufficient social support [7]. Neuroimaging studies have highlighted the relevance of the limbic system in the processing of perceived social support: Gray matter volumes of the posterior cingulate cortex [8] and amygdala [9] are positively associated with perceived social support. Brain functional studies in healthy adults have linked social support to decreased resting-state amygdala [10] and decreased insula activity, leading to fewer negative emotions during social exclusion [11].

CM, in contrast to social support, is a strong risk factor for various mental disorders, including major depressive disorder (MDD) [12**–**14] and post-traumatic stress disorder (PTSD) [15**–**17]. Research has shown opposite effects of social support and CM on mental health, potentially mediated through similar neural mechanisms. Healthy adolescents and adults with CM show reduced hippocampal volume [18**–**22] and increased limbic activity towards negative stimuli [19, 21, 23]. Notably, these neural changes linked to CM are already detectable in childhood [21, 24], emphasizing the importance of early years as a critical period for limbic development [25]. Further, there is evidence of distinct long-term neural consequences for different CM subtypes [26], though McCrory et al. [27] highlight the need for more research to differentiate between threat and deprivation types due to their co-occurrence. The observed neural patterns in individuals with CM mirror those found in patients with MDD and PTSD [28**–**33], suggesting that reduced limbic volume and limbic hyperresponsiveness may underpin the link between CM and the development of mental disorders [34, 35].

Despite the recognized influence of CM and perceived social support on mental health, there has been limited neuroimaging research exploring their combined effects on neural mechanisms. Previous studies demonstrated that regardless of the extent of experienced adversity, social support could mitigate the negative consequences of CM on limbic activity [36, 37] and developmental outcomes [38] across various life stages, including childhood [36], young adulthood [38] and later life [37]. Conversely, Luby et al. [39] suggest a buffering effect of social support on limbic structures irrespective of adverse life events only in pre-school children, but not in school-aged children. This suggests that early childhood is a pivotal period not only for the adverse effects of CM, which can result in lasting neural alterations in limbic structures into adulthood [21], but also for the buffering effects of social support and the potential for positive limbic development through caregiving [40, 41]. Beyond this critical period, limbic development may undergo irreversible changes due to CM, making later support less effective [39, 42]. Accordingly, evidence suggests that individuals with CM may exhibit diminished capacities for forming social connections, potentially leading to a persistent decrease in social support throughout later life [43**–**46]. This reduction in social ties could be attributed to the decreased benefits they perceive or receive from such relationships [47]. Hence, examining healthy adults with CM is crucial for understanding the enduring and possibly irreversible effects of CM, as well as the influence of current perceptions of social support on limbic structures, with reduced influence from ongoing brain development in critical developmental phases. Notably, on a structural level, Förster et al. [48] already showed that perceived social support had a protective effect on hippocampal volume in non-maltreated healthy adults only. Conversely, two recent studies revealed an inverse link between perceived social support and adverse experiences regarding structural brain connectivity [49] and white matter integrity [50] in adults, with the positive impacts of perceived social support manifesting independently of stressful life events [50].

Considering heterogeneous results across imaging modalities, it remains unclear whether buffering effects of social support on neural correlates of emotion processing are also present in healthy adults with CM. We assume that with transitioning from adolescence to adulthood, the source of social support shifts away from parents and caregivers towards a broader social network [51]. In the current study, we aimed to examine the relationship between perceived social support, CM, and limbic activity to negative facial stimuli in healthy adults. This approach allowed us to investigate the long-term consequences of CM on critical brain regions, without the potential confounding effects of mental health conditions and current clinical interventions, and with reduced influence from critical developmental changes typical in younger populations. Given the relevance of the hippocampus and amygdala for emotion processing [52] and their overlapping roles as neural correlates for CM and social support [39], we placed emphasis on these regions. Based on previous studies, we hypothesized a positive association of CM and a negative association of perceived social support with limbic activity during negative emotion processing. Furthermore, we expected CM to modulate the association between perceived social support and limbic activity.

## Materials and methods

### Participants and study design

All data of the present study are part of the Münster Neuroimaging Cohort (MNC) and represent original work. The study initially comprised 212 healthy individuals (HC) between 18 and 65 years of age, who were recruited through public notices and newspaper announcements from August 2013 to November 2019. All participants with complete data sets of functional magnetic resonance imaging (fMRI) as well as all relevant questionnaires (see below) were included. Exclusion criteria were any lifetime mental disorder according to the Structured Clinical Interview for DSM-IV (SCID-I) [53], any neurological abnormalities, a history of traumatic head injury, chronic medical diseases, organic mental disorders, dementia, intake of psychotropic medication or MRI contraindications. All participants completed fMRI, SCID-I [53], the Childhood Trauma Questionnaire (CTQ; [54]) and the Social Support Questionnaire (German version: Fragebogen sozialer Unterstützung, FSOZU-K-22; [55]). The CTQ is widely used to assess various forms of childhood trauma. The FSOZU-K-22 is used to evaluate an individual’s self-reported perceived social support (For psychometric information on the questionnaires, see Supplementary 1).

The sample (*n* = 212) was split into two groups based on cut-offs for CM established by Walker et al. [56]: HC with a score greater than or equal to the cut-off for CM on at least one subscale of the CTQ [54] were included in a group referred to as HC with CM *(*CM group*, n* = 65). HC scoring below the cut-offs on all CTQ subscales [54] were regarded as HC with no CM *(*nCM group*, n* = 147). There was a significant difference (T_*(106.335)*=_−2.476, *p* = 0.015, *d* = −0.393) in depressive symptom scores [57] between the nCM (*M* = 2.78, *SD* = 3.201) and CM group (*M* = 4.11, *SD* = 3.78). To rule out effects of subclinical depressive symptoms, the two groups were then matched in a 1:1 ratio based on the Beck Depression Inventory (BDI-I; [57, 58]; Supplementary 1) using the software MatchIt [59]. The final study sample consisted of *n* = 65 HC in the CM group and *n* = 65 HC in the nCM group. After matching, the groups showed no significant difference in BDI scores (Table 1). The research was conducted in accordance with the Helsinki Declaration as revised in 1989 and approved by the ethics committee of the Medical Faculty of University of Muenster (2007-307-f-S). All participants provided written informed consent before study participation and received a financial reimbursement. For details on sample characteristics, see Table 1.

**Table 1:** Sample characteristics.

	nCM group^a^ (*n* = 65)	CM group^a^ (*n* = 65)	*p* value^b^
*Sociodemographic characteristics*			
Age	45.72 ± 11.575	45.89 ± 11.521	0.934
% Female^c^	47.69	44.61	0.725^d^
*Questionnaires*			
BDI-I sum score	3.95 ± 3.573	4.11 ± 3.780	0.812
FSOZU-K-22 sum score	4.58 ± 0.416	4.33 ± 0.596	0.006
CTQ sum score	29.62 ± 3.404	39.66 ± 10.091	<0.001
Emotional abuse	29.62 ± 3.404	39.66 ± 10.091	<0.001
Physical abuse	5.89 ± 1.201	8.22 ± 3.595	0.002
Sexual abuse	5.22 ± 0.545	6.35 ± 2.825	0.011
Emotional neglect	5.08 ± 0.367	5.82 ± 2.256	<0.001
Physical neglect	8.02 ± 2.253	11.17 ± 4.471	<0.001
% above cut-off on CTQ subscales^c,e^			
Emotional abuse	0%	29.23%	–
Physical abuse	0%	20.00%	–
Sexual abuse	0%	13.85%	–
Emotional neglect	0%	24.62%	–
Physical neglect	0%	58.46%	–

*BDI* Beck depression inventory, *FSOZU-K-22* social support questionnaire, *CTQ* childhood trauma questionnaire, *NCM group* healthy individuals without childhood maltreatment, *CM group* healthy individuals with childhood maltreatment.

^a^Unless otherwise indicated, values are mean ± standard deviation of the sample.

^b^Unless otherwise indicated, *p*-values were obtained from an independent two-sample *t* test.

^c^Values describe percentages.

^d^*p*-value was derived from a *χ*^*2*^-test.

^e^According to Walker et al. [56].

### Functional MRI paradigm, data acquisition and preprocessing

Details on data acquisition, preprocessing methods and the fMRI paradigm can be found in Supplementary 2. Briefly, T2* functional data were acquired by a 3 Tesla scanner (Gyroscan Intera 3 T, Philips Medical Systems, Best, NL) and preprocessed using statistical parametric mapping software (SPM8, Wellcome Department of Cognitive Neurology, London, UK; http://www.fil.ion.ucl.ac.uk/spm). For the fMRI paradigm, a frequently applied [19, 20, 60] negative emotion processing task was employed, consisting of four blocks of a face-processing task with photographs of negative emotional faces (expressing fear or anger) and five blocks of a sensorimotor control task (geometric figures shaped as circles or ellipses).

### Statistical analyses

Clinical data was analyzed using SPSS Statistics (version 25.0; IBM Corporation). The Pearson correlation coefficient between CM and perceived social support was calculated for the final study sample (*N* = 130) to ascertain an opposing link between the two variables.

FMRI data analyses were performed by means of statistical parametric mapping software (SPM12, v7771, Wellcome Department of Cognitive Neurology, London, UK; http://www.fil.ion.ucl.ac.uk/spm). For all analyses in SPM, a region of interest (ROI) approach for the bilateral amygdala-hippocampus-complex (AHC) was conducted. One single ROI mask was created by means of the Wake Forest University PickAtlas [61] according to the AAL-atlas [62] definitions and included the mask of the bilateral amygdala and bilateral hippocampus. Additionally, age and sex were included as covariates of no interest. Significance thresholds for multiple testing were obtained at cluster-level by threshold-free cluster enhancement (TFCE) using the TFCE toolbox (version 232; Structural Brain Mapping Group, Jena, Germany; http://dbm.neuro.uni-jena.de/tfce). Results were considered significant if they exceeded a conservative FWE-corrected threshold of *p* < 0.05 obtained by 5000 permutations per test. The minimum cluster size was set at *k* ≥ 10 voxels.

In order to dimensionally investigate associations of perceived social support and CM on limbic activity and to verify previous findings, we first performed two separate regression analyses for the total score of the FSOZU-K-22 [55] and the total score of the CTQ [54] using the final study sample of 130 HC. Second, we conducted a social support (FSOZU-K-22 sum score) x group (CM vs. nCM group) analysis of covariance (ANCOVA) to investigate the interaction of perceived social support and CM on limbic activity. Main effects of group and social support as well as social support x group interaction effects on limbic activity were analyzed, followed by post-hoc tests. For exploratory reasons, we additionally performed the social support x group ANCOVA on whole-brain level using the same model. Here, a minimum cluster size of *k* ≥ 100 voxels was set.

## Results

### Association of childhood maltreatment with perceived social support

Perceived social support (FSOZU-K-22 sum score) and CM (CTQ sum score) showed a significant negative correlation (*r* = −0.293, *p* < 0.001) indicating that increased experience of CM was associated with lower perceived social support.

### Associations of childhood maltreatment and perceived social support with limbic activity

Higher CTQ scores were associated with higher limbic activity in bilateral clusters of the AHC during negative emotion processing (Left: x = −30, y = −28, z = −14, *TFCE*_*(126)*_ = 473.87, *T* = 4.66, *k* = 903, *p*_*FWE*_ = 0.006, *r* = 0.239; Right: x = 38, y = −28, z = −8, *TFCE*_*(126)*_ = 414.95, *T* = 4.66, *k* = 757, *p*_*FWE*_ = 0.009, *r* = 0.216). Higher perceived social support tended to be associated with lower left AHC activity (Left: x = −22, y = −12, z = −18, *TFCE*_*(126)*_ = 133.63, *T* = 3.59, *p*_*FWE*_ = 0.050, *r* = −0.136).

### Interaction of social support with limbic activity in maltreated vs. non-maltreated healthy individuals

**Fig. 1:**
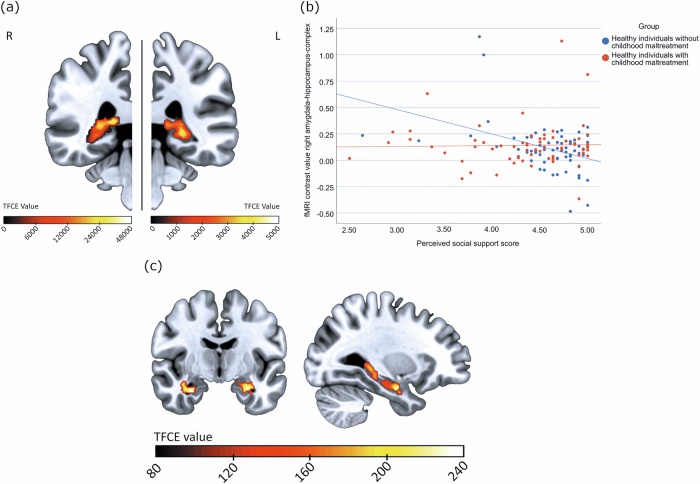
Association of perceived social support with limbic activity in response to negative emotional faces in dependence of childhood maltreatment. **a** Coronal view (Left and right: y = −36) of the significant social support x group interaction of the ANCOVA in the amygdala-hippocampus-complex (Left: x = −26, y = −36, z = −2, *TFCE*_*(1,124)*_ = 4033.90, *F* = 8.97, *k* = 217, *p*_*FWE*_ = 0.024, *η*_*p*_*²*=0.089 ; Right: x = 16, y = −36, z = 8, *TFCE*_*(1,124)*_ = 23703.43, *F* = 23.84, *k* = 646, *p*_*FWE*_ = 0.005, *η*_*p*_*²*=0.070). Color scale: *TFCE* value. Separate scales for each hemisphere for detailed resolution of the activity clusters. **b** Scatterplot depicts the association between activity within the right amygdala-hippocampus-complex derived from the social support x group interaction (x = 16, y = −36, z = 8, *TFCE*_*(1,124)*_ = 23703.43, *F* = 23.84, *k* = 646, *p*_*FWE*_ = 0.005, *η*_*p*_*²*=0.070) and perceived social support scores in healthy individuals without childhood maltreatment compared to healthy individuals with childhood maltreatment. **c** Coronal and sagittal view (x = −28, y = −8) of the significant negative association of perceived social support with functional activity within the amygdala-hippocampus-complex in the group without childhood maltreatment (Left: x = −28, y = −8, z = −24, *TFCE*_*(124)*_ = 231.28, *T* = 4.05, *k* = 504, *p*_*FWE*_ = 0.021, *r* = −0.429; Right: x = 14, y = −38, z = 8, *TFCE*_*(124)*_ = 325.67, *T* = 4.27, *k* = 591, *p*_*FWE*_ = 0.010, *r* = −0.411). Color scale: *TFCE* value.

The ANCOVA revealed a significant social support x group interaction on bilateral AHC activity (Left: x = −26, y = −36, z = −2, *TFCE*_*(1,124)*_ = 4033.90, *F* = 8.97, *k* = 217, *p*_*FWE*_ = 0.024, *η*_*p*_*²*=0.089 ; Left: x = −28, y = −8, z = −24, *TFCE*_*(1,124)*_ = 3066.00, *F* = 12.33, *k* = 62, *p*_*FWE*_ = 0.019, *η*_*p*_*²*=0.070; Right: x = 16, y = −36, z = 8, *TFCE*_*(1,124)*_ = 23703.43, *F* = 23.84, *k* = 646, *p*_*FWE*_ = 0.005, *η*_*p*_*²*=0.070; Fig. 1). This resulted from a significant negative association between perceived social support and bilateral limbic activity in the nCM group (Left: x = −28, y = −8, z = −24, *TFCE*_*(124)*_ = 231.28, *T* = 4.05, *k* = 504, *p*_*FWE*_ = 0.021, *r* = −0.429; Right: x = 14, y = −38, z = 8, *TFCE*_*(124)*_ = 325.67, *T* = 4.27, *k* = 591, *p*_*FWE*_ = 0.010, *r* = −0.411), with no significant association of perceived social support with limbic activity in the CM group (*p*_*FWE*_ = 0.420). There was also a significant main effect of perceived social support in the left AHC (x = −22, y = −12, z = −18, *TFCE*_*(1,124)*_ = 4517.64, *F* = 13.15, *k* = 107, *p*_*FWE*_ = 0.029) but no significant main effect of group (*p*_*FWE*_ > 0.99).

On whole-brain level, a significant social support x group interaction was found (all *p*_*FWE*_ ≤ 0.046, Supplementary 3, Table S1) in clusters comprising the hippocampus, parahippocampal gyrus, temporal gyri and amygdala. This resulted from a negative association between perceived social support and functional activity in the nCM group (all *p*_*FWE*_ ≤ 0.048), while there was no significant association of perceived social support with functional activity in the CM group (*p*_*FWE*_ = 0.464). There was no significant main effect of group (*p*_*FWE*_ > 0.99) or perceived social support (*p*_*FWE*_ ≥ 0.056) on whole-brain level.

### Robustness checks

Regression analyses incorporating either perceived social support or CM as covariates (Supplementary 4) and analyses in the unmatched sample of 147 HC without CM versus 65 HC with CM (Supplementary 5) confirmed the positive association of CTQ scores with AHC activity and a social support x group interaction on AHC activity. CM was dichotomized [56] to facilitate interpretability, enable intervention application, and allow comparison with a prior study [48]. However, given concerns about dichotomizing continuous variables [63] and female-based cut-offs [56], the main analysis using the continuous measure of CM was repeated. Results indicated that CM significantly moderated the link between perceived social support and limbic activity, corroborating previous findings (Supplementary 6, Fig. S1). Additional analyses with abuse versus neglect subtypes (Supplementary 6, Fig. S2) revealed that the moderation was only significant for childhood abuse, not neglect. Given the impact of perceived stress on the limbic system [64, 65], the influence of perceived stress indicating current stressful life events was also analyzed, with previous results remaining significant (Supplementry 7, Table S2, Fig. S3).

## Discussion

This study investigated the differential association of perceived social support with limbic activity in maltreated versus non-maltreated healthy adults during negative emotion processing. In HC without CM, increased perceived social support correlated with decreased limbic activity during processing of negative emotional faces, a link not observed in those with CM, even when controlling for perceived stress. Further analyses using continuous CM scores support these findings. Additionally, we confirmed that CM is positively associated with limbic activity to negative stimuli, independent of perceived social support, perceived stress and in the unmatched sample (*n* = 212), showing the robustness of our results.

### Interaction of social support with limbic activity in maltreated vs. non-maltreated healthy individuals

Previous studies highlight the pervasive psychological and physiological consequences of CM [16, 19, 43, 66–68], underscoring the major role of social support in mitigating stress and its adverse effects [6]. Our study focuses on the functional correlates of such interplay during negative emotion processing, revealing a significant influence on amygdala and hippocampus activity. We find that increased perceived social support correlates with decreased limbic activity during negative emotion processing in the nCM group, a link absent in the CM group. Further, continuous analyses also indicate that the extent of CM moderates the effect of perceived social support on limbic activity showing a negative trend in HC with low to moderate CM and, conversely, a positive trend in HC with high CM. With less CM, perceived social support serves as a protective buffer on limbic activity. Correspondingly, two studies have shown that the buffering effect of social support on mental health outcomes is more pronounced at lower levels of CM [69, 70]. Our finding seems to be driven by childhood abuse, as opposed to neglect. That is, higher abuse experiences seem to diminish the protective effect of perceived social support on limbic activity in healthy adults. This differential impact of abuse versus neglect aligns with the dimensional model of adversity and psychopathology, which suggests that abuse primarily disrupts emotion processing [71]. Conversely, another study observed that the detrimental impact of adversity on depressive symptoms decreased with more social support particularly in an abuse subgroup [72]. Our findings appear robust to the effects of current perceived stress, even though prior evidence suggests that stress is associated with heightened limbic activity during emotional face processing [73]. Additionally, our whole-brain analyses confirm a significant negative correlation in the nCM group, showing similar regions as the ROI analysis. Summarizing, in contrast to previous studies [10, 11], our results suggest that buffering effects of perceived social support on limbic activity are primarily effective in healthy adults with no or low to moderate CM, particularly abuse experiences, therewith extending previous studies [74**–**77].

The lack of a significant association between perceived social support and limbic activity in the CM group could be due to the profound impact of CM during a critical developmental period in childhood, leading to lasting neurobiological changes [78] like increased limbic activity. This could render perceived social support less effective as a buffer in adulthood. CM may alter social perception and information processing, potentially fostering threat sensitivity in social contexts, impaired social skills and confidence in receiving support [46, 79]. Accordingly, Maier et al. [80] observed that physical touch in individuals with CM elicited stress and increased limbic responses. While healthy adult individuals with CM may positively rate their social support upon conscious reflection, this support might act more as a stressor than a benefit due to an early negative bias towards emotion processing [48, 81]. In line, Hogan et al. [82] found that not all social support interventions are universally beneficial, with mismatches to individual needs potentially being detrimental. Our observed positive trend between perceived social support and limbic activity in HC with high CM levels, upon continuous analysis of CM, may also tentatively point to a detrimental effect of perceived social support. Additionally, our findings revealed lower levels of perceived social support in the CM group compared to the nCM group, also hinting at an altered perception of support. These findings, however, based on predominantly older adults without significant psychopathology, suggest a more resilient group and limit applicability to other groups. Despite being considered resilient, these adults seem to exhibit varied effects of perceived social support on limbic activity depending on levels of CM. Notably, however, research on resilience indicates that enhanced resilience can decrease the likelihood of adverse outcomes from CM [6, 35] and is associated with reduced limbic activity in response to negative stimuli following CM in adolescents as well as in adults [83, 84].

Wymbs et al. [36] found that social support moderated limbic activity during fearful face processing in children (7–16 years), regardless of child adversity, contrasting with our results of no such effect in healthy adults with CM. Besides the emotional identification task used by Wymbs et al. [36], which may explain the discrepancies in results, differences in the perceptions of social support during and after CM and its interaction with limbic activity could also play a role. Their study, assessing social support and CM using interviews and multiple data sources, evaluated social support closely following potential CM experiences indicating that social support may offer a protective effect during critical early periods. Accordingly, two recent studies have shown that in adolescents [39] and adults [48], a protective effect of support on hippocampal volume is apparent only in individuals without CM, supporting our findings. In contrast, in preschool children, a buffering effect was noted regardless of adverse life events [39]. Notably, our study is limited to examining the link between current perceived social support and limbic activity in dependence of earlier CM, without addressing perceptions of social support during CM itself. Interestingly, a previous study [85] suggests an association of childhood abuse with reduced perceived childhood social support, which appears to align with lower adult perceptions of social support. This tentatively supports a potential link between early and later perceptions of social support and their relation to CM.

The analysis showed no significant difference in limbic activity during negative emotion processing between groups. The categorization of participants into two groups based on a binary split of CM experiences led to heterogeneity within each group.

### Associations of childhood maltreatment and perceived social support with limbic activity

Using regression analyses to comprehensively explore the link between CM, perceived social support and limbic activity during negative emotion processing, we found that higher CM levels were associated with heightened limbic activity, even after accounting for perceived social support and perceived stress. This supports our first hypothesis and existing research [81, 86] showing enhanced limbic activity to negative emotions in healthy and clinical groups with CM. Such functional alterations are thought to be adaptive responses to stressful environments, enhancing sensitivity to negative cues [87, 88]. This heightened limbic activity in individuals with CM could potentially mediate the development of psychopathology [19, 21].

Perceived social support showed a trend towards reducing limbic activity during negative emotion processing, but this association became insignificant after adjusting for CM, likely due to a significant negative correlation between perceived social support and CM. While there is preliminary evidence suggesting social support can dampen limbic activity and enhance emotion regulation [10, 11], our methodology—lacking differentiation in social support types [11] and not examining spontaneous brain activity [10] or functional connectivity between limbic and frontal regions [8, 10]—may limit our findings. These findings imply that the neural impact of social support might be more complex than what was captured by our examination of limbic activity alone. Moreover, our results underline the moderating effect of CM on how perceived social support influences limbic activity, pointing to a multifaceted interaction between these factors.

### Strengths and limitations

Our study is the first to investigate the moderating effect of CM on the link between perceived social support and limbic activity during emotion processing in healthy adults, aged 18 to 65 years, without psychopathological conditions. This enabled the investigation of long-term effects of CM on the link between perceived social support and limbic activity in healthy adulthood. To avoid the confounding influences of mental health conditions, such as social withdrawal, symptom severity and interventions, we deliberately selected a healthy sample, despite acknowledging that including depressed individuals might have offered greater variance. This choice did not enable drawing conclusions about whether the identified mechanisms are similarly present in younger or psychiatric samples, affecting the generalizability of our findings to these groups. Our participants may represent a particularly resilient subgroup, having maintained their mental health despite past CM [35, 84]. Although our sample covered a wide age range that skewed towards older adulthood, and might therefore demonstrate greater psychological resilience compared to younger adults [89, 90], we still controlled for age in our analyses. Future studies should build upon our findings and explore the impact of perceived social support and CM on limbic responsivity in those with mental disorders and across different age groups in a comparative approach. While the questionnaires employed were reliable and validated, they assessed social support as perceived in adulthood, possibly not reflecting the perceived support experienced during CM. Future studies should also differentiate between perceived and actual social support changes by collecting data on received support. Moreover, we used the FSOZU-K-22 in its shortened form, which precluded a detailed analysis of specific subscales like emotional support, potentially relevant for emotion processing [11]. The CTQ, given its nature as a retrospective self-report questionnaire, is susceptible to negative recall biases. This may have affected the accuracy and objectivity of the reported experiences of CM, potentially skewing the interpretation of its effects on subsequent mental health outcomes. Nonetheless, empirical findings from a longitudinal study [91] highlight the CTQ’s temporal stability across various mood states, underscoring its effectiveness in reliably capturing CM reports over time. To enhance the precision of CM assessment, future research should, however, also consider more detailed measures of CM, including the frequency, timing, and subtypes of maltreatment experiences. Additionally, the binary categorization of participants based on CTQ scores led to information loss and group heterogeneity. Moreover, the cut-offs used [56], derived from an exclusively female sample, might not be appropriately tailored for our mixed-gender sample. This could lead to biased results, particularly given well-documented sex differences in reporting CM [92]. Nevertheless, additional analyses treating CTQ and FSOZU-K-22 scores as dimensional provided comparable results, supporting the robustness of our conclusions. Finally, given the cross-sectional design of our study, we cannot establish causal relationships from our data.

### Conclusion and implications

Extending previous studies, our findings highlight how CM influences the relationship between perceived social support and limbic activity during negative emotion processing in healthy adults, even after accounting for depressive symptoms and perceived stress. While perceived social support may mitigate limbic activity in healthy adults without (or low to moderate levels of) CM, this protective effect is less evident in those with (high levels of) CM. In this context, abuse experiences appear to be particularly relevant. Notably, healthy adults with CM generally perceive less social support, together pointing to the need for interventions to improve social support perception [48, 93], coping strategies, and self-esteem [94] in those affected by CM. Such interventions could reshape early social experiences and perceptions, potentially reducing psychopathological risk.

## Supplementary information

Supplementary Material

## Data Availability

The data of this study are available on reasonable request from the corresponding author. The data are not publicly available due to privacy or ethical restrictions.
