# Supplementary material for: Interaction of perceived social support and childhood maltreatment on limbic responsivity towards negative emotional stimuli in healthy individuals
Source: Neuropsychopharmacology. 2024 Jul 1;49(11):1775–82. doi: 10.1038/s41386-024-01910-6 (PMC11399403; doi:10.1038/s41386-024-01910-6)
Supplement: Supplementary file 1 — Supplementary Material [file 41386_2024_1910_MOESM1_ESM.doc]

Supplementary material

[Supplement 1: Questionnaires 2](#__RefHeading___Toc165114455)

[Supplement 2: Functional MRI paradigm, data acquisition and preprocessing 3](#__RefHeading___Toc165114456)

[Data acquisition and preprocessing 3](#__RefHeading___Toc165114457)

[Face matching paradigm 4](#__RefHeading___Toc165114458)

[Supplement 3: Results of the social support x group interaction on whole-brain level 5](#__RefHeading___Toc165114459)

[Supplement 4: Robustness check with CTQ or FSOZU-K-22 as a covariate 6](#__RefHeading___Toc165114460)

[Supplement 5: Analyses in the unmatched sample 7](#__RefHeading___Toc165114461)

[Association of childhood maltreatment and perceived social support with limbic activity 7](#__RefHeading___Toc165114462)

[Interaction of social support with limbic activity in 65 maltreated vs. 147 non-maltreated healthy individuals 7](#__RefHeading___Toc165114463)

[Supplement 6: Analyses using the continuous measure of childhood maltreatment 8](#__RefHeading___Toc165114464)

[Childhood maltreatment as the moderator of the perceived social support – limbic activity association 9](#__RefHeading___Toc165114465)

[Further analyses with the abuse versus neglect subtypes of childhood maltreatment 9](#__RefHeading___Toc165114466)

[Supplement 7: Current perceived stress 11](#__RefHeading___Toc165114467)

[Association of childhood maltreatment with limbic activity using perceived stress as a covariate 11](#__RefHeading___Toc165114468)

[Association of social support with limbic activity using perceived stress as a covariate 12](#__RefHeading___Toc165114469)

[Interaction of social support with limbic activity in maltreated vs. non-maltreated healthy individuals using perceived stress as a covariate 12](#__RefHeading___Toc165114470)

[Analyses using the continuous measure of childhood maltreatment with perceived stress as a covariate 14](#__RefHeading___Toc165114471)

[References 16](#__RefHeading___Toc165114472)

## **Supplement 1: Questionnaires**

Information on childhood maltreatment (CM) was gathered by means of the German version of the Childhood Trauma Questionnaire (CTQ) [1,2]. The CTQ [2] consists of 28 items, subcategorized in five subscales (emotional abuse, physical abuse, sexual abuse, emotional neglect, and physical neglect) with five items each, and three items intending to measure possible trivialization by the victim. The internal consistency of all subscales is moderate to high with Cronbach’s α≥0.80 [1]. Cut-off scores established by Walker et al. [3] are the following: sexual abuse (≥ 8), physical abuse (≥8), emotional abuse (≥10), emotional neglect (≥15) and physical neglect (≥8). These cut-off scores had a sensitivity and specificity of ≥0.85 on all five subscales [3]. Participants meeting criteria for at least one cut-off on one subscale are considered healthy individuals (HC) with CM. When considering the continuous measure of the CTQ, a higher score indicates a greater extent of experienced childhood maltreatment [2].

Social support can be measured in terms of either perceived or received social support, with evidence suggesting that perceived social support, in particular, has a positive effect on developmental outcomes following exposure to CM [4]. We measured perceived social support using the German version of the 22-item short version of the Social Support Questionnaire (FSOZU-K-22) [5,6]. The FSOZU-K-22 [6] has high internal consistency (Cronbach’s α in a general population sample α = 0.87 and in a clinical sample α = 0.96 [7], and in another representative German population sample α = 0.81-0.93 [5]). According to Dunkel et al. [7], the 22-item short version does not allow for analysis on subscale level but only on the total score level. Therefore, we used the total score for our analyses with a higher score indicating higher overall perceived social support.

The German version of the Beck Depression Inventory (BDI-I) [8,9] was used to further evaluate self-reported depressive symptoms on a subclinical level. In previous studies, an internal consistency of usually α ≥ 0.75 was found [10].

## **Supplement 2: Functional MRI paradigm, data acquisition and preprocessing**

### **Data acquisition and preprocessing**

T2* functional data were acquired by a 3 Tesla scanner (Gyroscan Intera 3T, Philips Medical Systems, Best, NL) using a single-shot echoplanar sequence, with parameters selected to minimize distortion in the region of central interest, while retaining adequate signal-to- noise ratio (S/N) and T2* sensitivity. Volumes were acquired in 34 slices (matrix 64 X 64, resolution 3.6 X 3.6 X 3.6mm, repetition time = 2.1s, echo time = 30ms, flip angle = 90°). To minimize susceptibility-related dropout artefacts in the orbitofrontal and mediotemporal regions, slices were tilted 25° from the anterior commissure/posterior commissure line.

Preprocessing of fMRI data was performed by means of statistical parametric mapping software (SPM8, Wellcome Department of Cognitive Neurology, London, UK; <http://www.fil.ion.ucl.ac.uk/spm>) and included realignment, unwarping, spatially normalizing of each participant’s functional images to the Montreal Neurological Institute International Consortium (MNI) for Brain Mapping template and smoothing (Gaussian kernel, 6 mm full-width at half maximum). The onsets and durations of the experimental conditions (faces and shapes) were modeled using a canonical hemodynamic response function in the context of a general linear model. The model was corrected for serial correlations. A high-pass filter of 128s was used to remove low-frequency noise. For each participant, one contrast image was generated in each individual first-level analysis (faces > shapes) to compare activation during negative face processing versus shape processing.

### **Face matching paradigm**

Functional magnetic resonance imaging (fMRI) was used to measure emotion processing responses in the limbic system. We conducted a robust paradigm that has been used in various studies [11–13]. It consists of four blocks of a face-processing task and five blocks of a sensorimotor control task. In the face-processing task, three faces expressing either anger or fear were shown, two of which were identical. Participants were instructed to match the identical one of two faces on the bottom to the picture on the top. Each block consisted of six face trios presented for 4 seconds, separated by a variable interstimulus interval of 3.5±2 seconds. Fixed total block duration was 45 seconds. Faces were obtained from the Ekman and Friesen stimulus set [14]. The images of each block were balanced for gender and emotion. To eliminate primary sensorimotor signals from the face-matching task [15] a sensorimotor control task was used as control condition. The task consisted of geometrical shapes (circles and ellipses) that had to be matched in a similar way. Each of the five sensorimotor control blocks consisted of six different shape trios presented for 4 seconds and separated by a fixed interstimulus interval of 1.5 seconds, resulting in a duration of 33 seconds. Task duration in total was 363 seconds. The accuracy and response time of participants was measured.

## **Supplement 3: Results of the social support x group interaction on whole-brain level**

| **Table S1.** Results of the social support x group interaction on whole-brain level | | | | | | | | |
| --- | --- | --- | --- | --- | --- | --- | --- | --- |
| Anatomical region | Side | Cluster size^1^ | Peak voxel coordinates | | | Test statistics | | |
|  |  |  | x | y | z |  |  |  |
| **Group x time interaction effect^2^** | | | | | | *F-*value | *TFCE*-value | *p_FWE_*-value |
| Hippocampus/ precuneus/ parahippocampal gyrus (g.)/ middle temporal g./ fusiform g./ posterior cingulate g./ lingual g./ inferior temporal g./ amygdala | L/R | 2628 | 16 | -36 | 8 | 23.84 | 46214.68 | .028 |
| Hippocampus/ fusiform g./ parahippocampal g./ cerebellum/ thalamus | L | 748 | -28 | -44 | 8 | 21.25 | 32589.30 | .034 |
| Fusiform g./ lingual g./ cerebellum | R | 155 | 30 | -68 | -14 | 22.49 | 32514.50 | .034 |
| Lingual g./ cerebellum/ calcarine fissure | L | 228 | -10 | -46 | -6 | 18.26 | 25736.18 | .039 |
| Postcentral g./ rolandic operculum/ precentral g. | R | 502 | 64 | 0 | 22 | 17.03 | 23863.89 | .041 |
| Middle temporal g./ angular g./ superior temporal g. | R | 120 | 44 | -54 | 16 | 16.66 | 23752.07 | .041 |
| Caudate nucleus | L | 225 | -14 | 14 | 14 | 15.51 | 22902.07 | .042 |
| Insula/ rolandic operculum/ heschl g. | R | 112 | 40 | -16 | 18 | 11.00 | 19516.70 | .046 |
| **Post-hoc tests^3^** |  |  |  |  |  | *T-*value |  |  |
| *Negative association in nCM group* | | | | | | | | |
| Hippocampus/ precuneus/ fusiform g./ parahippocampal g./ posterior cingulate g./ insula/ rolandic operculum/ lingual g./ postcentral g./ inferior temporal g./ middle temporal g. | L/R | 9166 | 2 | -38 | 18 | 5.69 | 1792.77 | .022 |
| Middle frontal g./ superior frontal g. | L | 523 | -24 | 20 | 36 | 4.07 | 1283.61 | .042 |
| Caudate nucleus | L/R | 932 | -8 | 4 | 20 | 3.83 | 1271.10 | .043 |
| Superior frontal g./ anterior cingulate g. | L/R | 225 | -4 | 48 | 32 | 3.56 | 1192.01 | .048 |
| *Positive association in CM group* | | | | | | | | |
|  | - | - | - | - | - | - | - | .464 |
| *Abbreviations.* NCM group, Healthy individuals without childhood maltreatment; CM group, Healthy individuals with childhood maltreatment. ^1^ The table shows clusters with *p_FWE_* < .05, a minimum cluster size *k* ≥ 100 and brain regions that were represented in the cluster with more than 2% of their total size. ^2^ df_1_= 1; df_2_=124. ^3^ df=124. | | | | | | | | |

## **Supplement 4: Robustness check with CTQ or FSOZU-K-22 as a covariate**

With perceived social support as a covariate, the regression analysis of the CTQ still revealed that higher CTQ scores were associated with increased limbic activity in bilateral clusters of the AHC during the processing of negative emotional faces (Left: x=-30, y=-28, z=-14, *TFCE_(125)_*=375.57, *T*=4.40, *k*=645, *p_FWE_*=.012, *r*=.237; Right: x=38, y=-28, z=-8, *TFCE_(125)_*=340.10, *T*=4.55, *k*=445, *p_FWE_*=.015, *r*=.212). When controlling for CM, there was no significant association between perceived social support and AHC activity (*p_FWE_*≥.124).

## **Supplement 5: Analyses in the unmatched sample**

### **Association of childhood maltreatment and perceived social support with limbic activity**

The regression analysis showed that higher CTQ scores were associated with higher limbic activity in bilateral clusters of the AHC during negative emotional face processing (Left: x=-30, y=-32, z=-12, *TFCE_(208)_*=460.93, *T*=4.27, *k*=910, *p_FWE_*=.004, *r*=.459; Right: x=36, y=-40, z=-6, *TFCE_(208)_*=414.95, *T*=3.60, *k*=549, *p_FWE_*=.004, *r*=.357). There was no significant association of perceived social support with AHC activity (*p_FWE_*=.132).

### **Interaction of social support with limbic activity in 65 maltreated vs. 147 non-maltreated healthy individuals**

There was a significant social support x group interaction on right AHC activity (Right: x=34, y=-22, z=-14, *TFCE_(1,206)_*=5080.66, *F*=14.96, *k*=66, *p_FWE_*=.021; Right: x=16, y=-36, z=8, *TFCE_(1,206)_*=3301.58, *F*=13.62, *k*=22, *p_FWE_*=.029; Right: x=28, y=-34, z=-4, *TFCE_(1,206)_*=1753.48, *F*=9.04, *k*=10, *p_FWE_*=.045). The post-hoc tests revealed only a tendency for a negative association between social support and limbic activity in the nCM group (*p_FWE_*=0.071), while there was no significant association of perceived social support with limbic activity in the CM group (*p_FWE_*=.428). There was no main effect of group or perceived social support (both *p_FWE_*>.99).

## **Supplement 6: Analyses using the continuous measure of childhood maltreatment**

As described in the manuscript, we applied cut-offs based on Walker et al. [3] in order to provide comparable results to those of a previous study [16]. However, given concern regarding the dichotomization of continuous variables [17], and the derivation of used cut-offs based on an exclusively female sample, we repeated the main analysis investigating the interaction of perceived social support and CM on AHC activity using the continuous metric of the CTQ. Additionally, we conducted this analysis separately for the subtypes of abuse and neglect. This aimed to consider the potentially varying impacts of these subtypes on the association between social support and limbic activity. The subscore for abuse was created by summing the three subscales for abuse from the CTQ (emotional abuse, physical abuse, and sexual abuse; [2]), while the subscore for neglect was created by summing the two subscales for neglect (emotional neglect, physical neglect). In all analyses, significant clusters of the interaction term were extracted to SPSS Statistics (version 25.0; IBM Corporation) for graphical illustration. For all analyses in SPM, a region of interest (ROI) approach for the bilateral AHC was conducted. One single ROI mask was created by means of the Wake Forest University PickAtlas [18] according to the AAL-atlas [19] definitions and included the mask of the bilateral amygdala and bilateral hippocampus. Age, sex and BDI-I were used as covariates. Significance thresholds for multiple testing were obtained at cluster-level by TFCE using the TFCE toolbox (version 232; Structural Brain Mapping Group, Jena, Germany; http://dbm.neuro.uni-jena.de/tfce). Results were considered significant if they exceeded a conservative FWE-corrected threshold of p<.05 obtained by 5000 permutations per test. The minimum cluster size was set at k ≥ 10 voxels.

### **Childhood maltreatment as the moderator of the perceived social support – limbic activity association**

There was a significant social support x CM interaction on left AHC activity (Left: x=-32, y=-40, z=-2, *TFCE_(1,205)_*=182.67, *T*=4.16, *k*=66, *p_FWE_*=.025) showing that the continuous measure of CM significantly moderated the link between perceived social support and limbic activity (**Figure S1**).


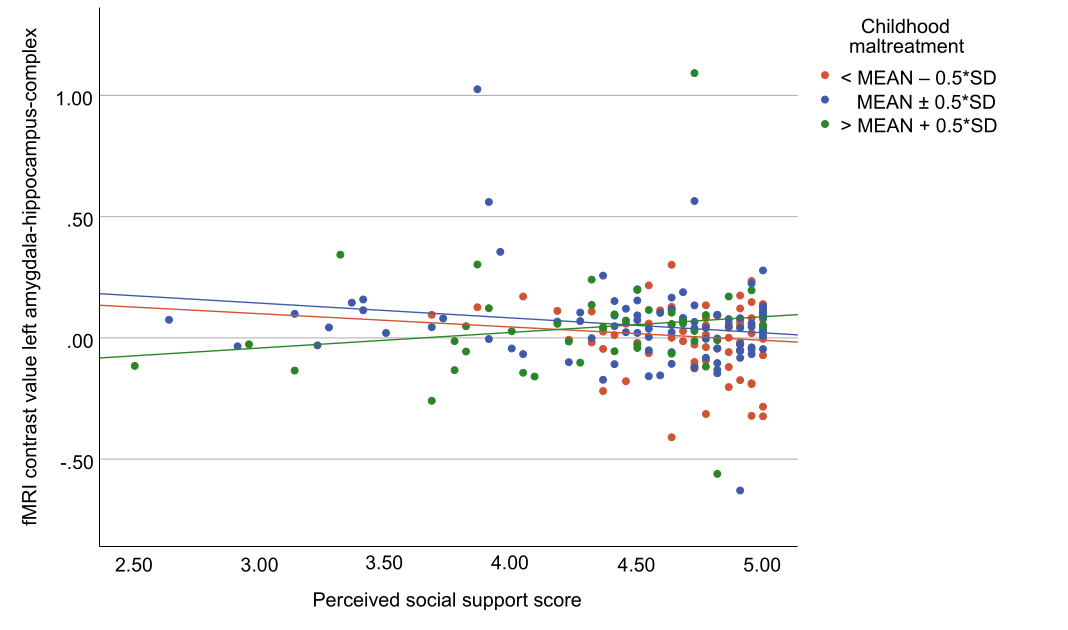

**Figure S1.** **Moderating effect of childhood maltreatment on the association between perceived social support and limbic activity in the full sample.** The continuous measure of childhood maltreatment was discretized for illustration purposes only. The discretization was performed based on the standard deviation (SD) to address the variability in the data. A half SD was chosen for finer segmentation to identify more subtle differences in the data. Age, sex and BDI-I were used as covariates.

### **Further analyses with the abuse versus neglect subtypes of childhood maltreatment**

For the subscore childhood abuse, the regression analysis yielded a significant social support x childhood abuse interaction on left AHC activity (Left: x=-32, y=-42, z=-4, *TFCE_(1,205)_*=184.46, *T*=4.09, *k*=32, *p_FWE_*=.033). This indicates that the continuous measure of childhood abuse significantly moderated the link between perceived social support and limbic activity (**Figure S2**). Contrary, there was no significant interaction between perceived social support and childhood neglect on AHC activity (*p_FWE_*=.066).


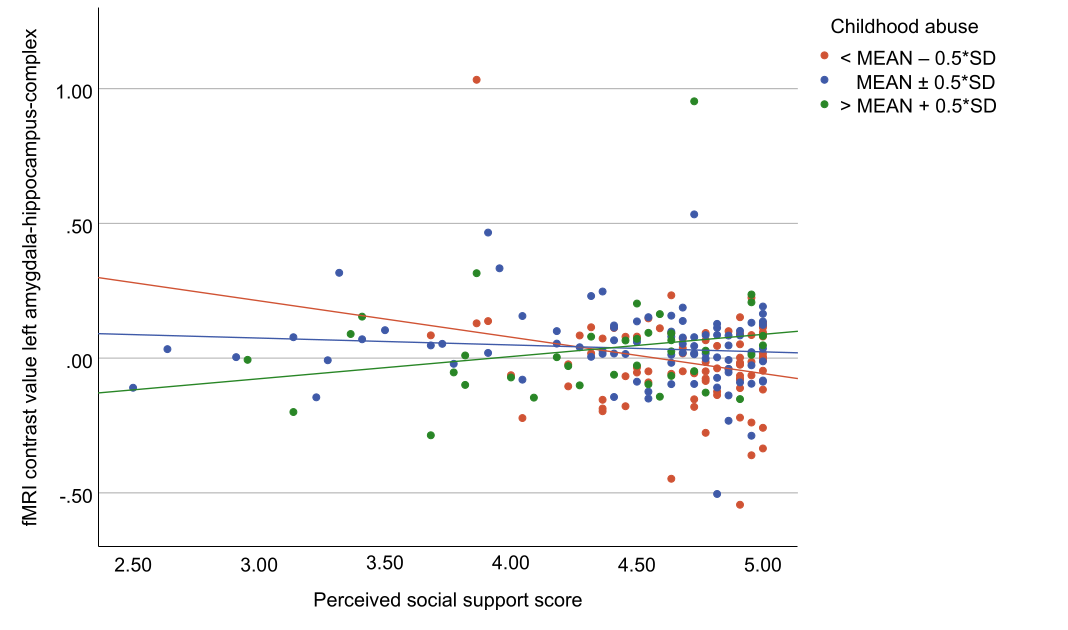

**Figure S2.** **Moderating effect of childhood abuse on the association between perceived social support and limbic activity in the full sample.** The continuous measure of childhood abuse was discretized for illustration purposes only. The discretization was performed based on the standard deviation (SD) to address the variability in the data. A half SD was chosen for finer segmentation to identify more subtle differences in the data. The subscore for childhood abuse was created by summing the three subscales for abuse from the CTQ (emotional abuse, physical abuse, and sexual abuse). Age, sex and BDI-I were used as covariates.

## **Supplement 7: Current perceived stress**

The German version of the 14-item perceived stress scale (PSS-14) was utilized as an indicator for current life stressors [20]. The PSS-14 assesses stressors, their subjective evaluations, and stress reactions within the last month, with higher scores indicating greater stress experience. In previous studies, an internal consistency of α ≥ 0.70 was found [21].

Using Pearson correlation coefficient in SPSS Statistics (version 25.0; IBM Corporation), perceived stress (PSS-14 sum score) showed a significant negative correlation (*r*=-.332, *p*<.001) with perceived social support (FSOZU-K-22 sum score) indicating that an increased perception of stress was associated with lower perceived social support. There was no significant association (*p*=.133) of perceived stress with CM (CTQ sum score) or with AHC activity during negative emotion processing (*p_FWE_*=.439). Still, to account for the influence of perceived stress in our analyses, we repeated all analyses with the PSS-14 as an additional covariate. Since we applied the same statistical methods for these analyses, we will only present the corresponding results here.

### **Association of childhood maltreatment with limbic activity using perceived stress as a covariate**

The positive association of CTQ scores with limbic activity in bilateral clusters of the AHC during negative emotion processing remained significant when controlling for perceived stress (Left: x=-30, y=-28, z=-14, *TFCE_(125)_*=477.89, *T*=4.74, *k*=865, *p_FWE_*=.007, *r*=.364; Right: x=38, y=-28, z=-8, *TFCE_(125)_*=428.69, *T*=3.73, *k*=746, *p_FWE_*=.009, *r*=.306).

### **Association of social support with limbic activity using perceived stress as a covariate**

With perceived stress as a covariate, the regression analysis of the FSOZU-K-22 revealed that higher perceived social support was associated with decreased limbic activity in the left AHC during the processing of negative emotional faces (Left: x=-22, y=-14, z=-20, *TFCE_(125)_*=149.06, *T*=3.52, *k*=13, *p_FWE_*=.012, *r*=-.229).

### **Interaction of social support with limbic activity in maltreated vs. non-maltreated healthy individuals using perceived stress as a covariate**

When controlling for perceived stress, the ANCOVA still revealed a significant social support x group interaction on the activity of two clusters in the right AHC (Right: x=16, y=-36, z=8, *TFCE_(1,123)_*=24814.36, *F*=23.72, *k*=391, *p_FWE_*=.015; Right: x=16, y=-38, z=-8, *TFCE_(1,123)_*=12677.25, *F*=18.53, *k*=46, *p_FWE_*=.032). This resulted from a significant negative association between perceived social support and bilateral limbic activity in the nCM group (Left: x=-32, y=-26, z=-22, *TFCE_(123)_*=430.21, *T*=4.18, *k*=1109, *p_FWE_*=0.011, *r*=-.326; Right: x=28, y=-34, z=-4, *TFCE_(123)_*=318.80, *T*=4.11, *k*=692, *p_FWE_*=0.021, *r*=-.314), while there was no significant association of perceived social support with limbic activity in the CM group (*p_FWE_*=.306). There was also a significant main effect of perceived social support in two clusters of the left AHC during negative emotional face processing (Left: x=-18, y=-20, z=-26, *TFCE_(1,123)_*=6321.55, *F*=12.86, *k*=56, *p_FWE_*=.042; Left: x=-32, y=-26, z=-22, *TFCE_(1,123)_*=6132.07, *F*=14.59, *k*=15, *p_FWE_*=.043) but no significant main effect of group (*p_FWE_*>.99).

On whole-brain level, a significant social support x group interaction was found (all *p_FWE_*≤.040, **Table S2**) in clusters comprising the hippocampus, precuneus, parahippocampal gyrus and temporal gyri. This effect was driven by a negative association between perceived social support and functional activity in the nCM group (all *p_FWE_*≤.046), while there was no significant association within the CM group (*p_FWE_*=.556). There was no significant main effect of group (*p_FWE_*>.99) or perceived social support (*p_FWE_*≥.061) on whole-brain level in clusters ≥ 100 voxels.

| **Table S2.** Results of the social support x group interaction on whole-brain level with perceived stress as a covariate | | | | | | | | |
| --- | --- | --- | --- | --- | --- | --- | --- | --- |
| Anatomical region | Side | Cluster size^1^ | Peak voxel coordinates | | | Test statistics | | |
|  |  |  | x | y | z |  |  |  |
| **Group x time interaction effect^2^** | | | | | | *F-*value | *TFCE*-value | *p_FWE_*-value |
| Hippocampus/ postcentral gyrus (g.)/ precuneus/ rolandic operculum/ parahippocampal g./ middle temporal g./ insula/ posterior cingulate cortex/ fusiform g./ precentral g./ lingual g./ cerebellum | L/R | 6494 | 16 | -36 | 8 | 23.72 | 47190.03 | .025 |
| Fusiform g./ lingual g./ cerebellum | R | 230 | 30 | -68 | -14 | 22.03 | 30647.68 | .032 |
| Superior frontal g./ middle frontal g. | R | 368 | 22 | 40 | 40 | 20.80 | 29718.76 | .032 |
| Middle temporal g./ angular g./ middle occipital g. | L | 370 | -50 | -70 | 38 | 22.33 | 27994.47 | .033 |
| Caudate nucleus/ thalamus/ | L/R | 1201 | -14 | 14 | 14 | 16.28 | 25539.21 | .035 |
| Middle temporal g./ angular g./ superior temporal g. | R | 230 | 44 | -54 | 16 | 16.71 | 22546.36 | .038 |
| Superior frontal g./ middle frontal g./ supracallosal anterior cingulate cortex/ middle cingulate cortex | L | 646 | -28 | 34 | 46 | 14.33 | 21310.80 | .040 |
| **Post-hoc tests^3^** |  |  |  |  |  | *T-*value |  |  |
| *Negative association in nCM group* | | | | | | | | |
| Precuneus/ hippocampus/ para-hippocampal g./ post-central g./ rolandic operculum/ insula/ posterior cingulate cortex/ fusiform g./ superior temporal g./ middle temporal g./ lingual g. | L/R | 10632 | 2 | -38 | 18 | 6.13 | 1940.52 | .003 |
| Superior frontal g./ caudate nucleus/ middle frontal g./ pregenual anterior cingulate cortex/ supracallosal anterior cingulate cortex/ middle cingulate cortex | L/R | 5719 | 24 | 40 | 38 | 4.60 | 1404.01 | .006 |
| Fusiform g./ lingual g. | R | 100 | 30 | -68 | -14 | 4.97 | 1294.91 | .001 |
| Middle temporal g./ angular g./ middle occipital g. | L | 426 | -50 | -70 | 38 | 4.74 | 1277.84 | .001 |
| Postcentral g./ precentral g. | L | 228 | -54 | -6 | 30 | 3.80 | 1223.83 | .003 |
| Inferior frontal g. | R | 291 | 46 | 26 | 8 | 3.64 | 1215.34 | .003 |
| *Positive association in CM group* | | | | | | | | |
|  | - | - | - | - | - | - | - | .556 |
| *Abbreviations.* NCM group, Healthy individuals without childhood maltreatment; CM group, Healthy individuals with childhood maltreatment. ^1^ The table shows clusters with *p_FWE_* < .05, a minimum cluster size *k* ≥ 100 and brain regions that were represented in the cluster with more than 2% of their total size. ^2^ df_1_= 1; df_2_=123. ^3^ df=123. | | | | | | | | |

### **Analyses using the continuous measure of childhood maltreatment with perceived stress as a covariate**

With perceived stress as a covariate, the social support x CM interaction on bilateral AHC activity remained significant (Left: x=-32, y=-40, z=-2, *TFCE_(1,204)_*=192.13, *T*=4.18, *k*=101, *p_FWE_*=.020; Right: x=32, y=-22, z=-14, *TFCE_(1,204)_*=182.21, *T*=3.88, *k*=83, *p_FWE_*=.023; Right: x=18, y=-30, z=-14, *TFCE_(1,204)_*=150.51, *T*=3.60, *k*=18, *p_FWE_*=.038; **Figure S3**).


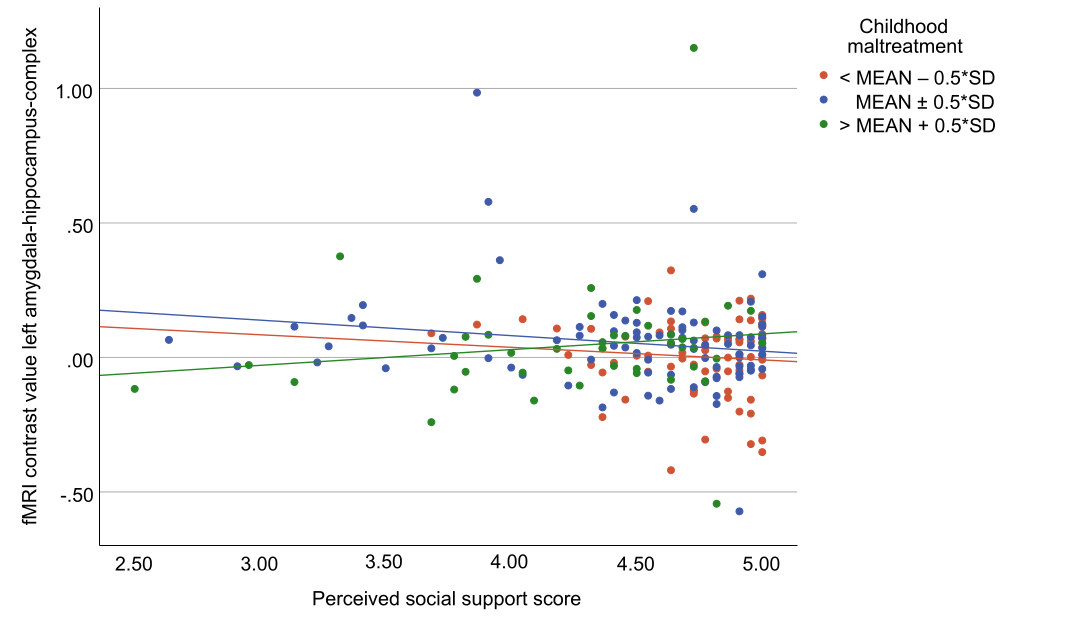
**Figure S3.** **Moderating effect of childhood maltreatment on the association between perceived social support and limbic activity in the full sample, with perceived stress as an additional covariate.** The continuous measure of childhood maltreatment was discretized for illustration purposes only. The discretization was performed based on the standard deviation (SD) to address the variability in the data. A half SD was chosen for finer segmentation to identify more subtle differences in the data. Age, sex, BDI-I and PSS-14 were used as covariates.

## **References**

1. Klinitzke G, Romppel M, Häuser W, Brähler E, Glaesmer H. The German Version of the Childhood Trauma Questionnaire (CTQ): psychometric characteristics in a representative sample of the general population. Psychother Psychosom Med Psychol. 2012;62:47–51.

2. Wingenfeld K, Spitzer C, Mensebach C, Grabe HJ, Hill A, Gast U, et al. The German version of the Childhood Trauma Questionnaire (CTQ): Preliminary psychometric properties. Psychother Psychosom Med Psychol. 2010;60:442–450.

3. Walker EA, Gelfand A, Katon WJ, Koss MP, Korff MV, Bernstein D, et al. Adult Health Status of Women with Histories of Childhood Abuse and Neglect. Am J Med. 1999;107:332–339.

4. Pepin EN, Banyard VL. Social Support: A Mediator between Child Maltreatment and Developmental Outcomes. J Youth Adolesc. 2006;35:612–625.

5. Brähler E, Geyer M, Homas Fydrich T. Fragebogen zur Sozialen Unterstützung (F-SozU): Normierung an einer repräsentativen Stichprobe. Diagnostica. 1999;45:212–216.

6. Frydrich T, Sommer G, Brähler E. Fydrich, T., Sommer, G., & Brähler, E. (2007). Fragebogen zur Sozialen Unterstützung: F-SozU; Manual. Hogrefe. 2007.

7. Dunkel D, Antretter E, Fröhlich-Walser S, Haring C. Evaluation of the Short-Form Social Support Questionnaire (SOZU-K-22) in Clinical and Non-Clinical Samples. Psychother Psych Med. 2005;55:266–277.

8. Beck AT, Steer RA, Brown GK. Beck Depression Inventory. New York: Harcourt Brace Jovanovich; 1987.

9. Hautzinger M. Das Beck-Depressioninventar (BDI) in der Klinik [The German version of the Beck Depression Inventory (BDI) in clinical use]. Nervenarzt. 1991;62:689–696.

10. Beck AT, Steer RA, Carbin MC. Psychometric properties of the beck depression inventory: Twenty-five years of evaluation. Clin Psychol Rev. 1988;8:77–100.

11. Dannlowski U, Stuhrmann A, Beutelmann V, Zwanzger P, Lenzen T, Grotegerd D, et al. Limbic scars: Long-term consequences of childhood maltreatment revealed by functional and structural magnetic resonance imaging. Biol Psychiatry. 2012;71:286–293.

12. Enneking V, Dzvonyar F, Dück K, Dohm K, Grotegerd D, Förster K, et al. Brain functional effects of electroconvulsive therapy during emotional processing in major depressive disorder. Brain Stimulat. 2020;13:1051–1058.

13. Redlich R, Grotegerd D, Opel N, Kaufmann C, Zwitserlood P, Kugel H, et al. Are you gonna leave me? Separation anxiety is associated with increased amygdala responsiveness and volume. Soc Cogn Affect Neurosci. 2015;10:278–284.

14. Ekman P, Friesen WV. Pictures of facial affect. Palo Alto. 1976.

15. Tessitore A, Hariri AR, Fera F, Smith WG, Das S, Weinberger DR, et al. Functional changes in the activity of brain regions underlying emotion processing in the elderly. Psychiatry Res - Neuroimaging. 2005;139:9–18.

16. Förster K, Danzer L, Redlich R, Opel N, Grotegerd D, Leehr EJ, et al. Social support and hippocampal volume are negatively associated in adults with previous experience of childhood maltreatment. J Psychiatry Neurosci. 2021;46:E328–E336.

17. MacCallum RC, Zhang S, Preacher KJ, Rucker DD. On the practice of dichotomization of quantitative variables. Psychol Methods. 2002;7:19–40.

18. Maldjian JA, Laurienti PJ, Kraft RA, Burdette JH. An automated method for neuroanatomic and cytoarchitectonic atlas-based interrogation of fMRI data sets. NeuroImage. 2003;19:1233–1239.

19. Tzourio-Mazoyer N, Landeau B, Papathanassiou D, Crivello F, Etard O, Delcroix N, et al. Automated anatomical labeling of activations in SPM using a macroscopic anatomical parcellation of the MNI MRI single-subject brain. NeuroImage. 2002;15:273–289.

20. Cohen S, Kamarck T, Mermelstein R. A Global Measure of Perceived Stress. J Health Soc Behav. 1983;24:385–396.

21. Lee E-H. Review of the Psychometric Evidence of the Perceived Stress Scale. Asian Nurs Res. 2012;6:121–127.
